# PCSK9 inhibitors for acute coronary syndrome: the era of early implementation

**DOI:** 10.3389/fcvm.2023.1138787

**Published:** 2023-05-02

**Authors:** Hongzhen Chen, Xiaomin Chen

**Affiliations:** Ningbo First Hospital, Ningbo, China

**Keywords:** PCSK9 inhibitors, acute coronary syndrome, coronary heart disease, early implementation, low-density lipoprotein cholesterol

## Abstract

Proprotein convertase subtilisin/kexin type 9 (PCSK9) inhibitors, a new cholesterol-lowering strategy, can decrease low-density lipoprotein cholesterol (LDL-C) levels by inhibiting PCSK9 and reducing the degradation of LDL receptors; thus, they are impacting the management of dyslipidemia to the prevention of cardiovascular events. Recent guidelines recommend PCSK9 inhibitors for patients who fail to achieve target lipids after ezetimibe/statin therapy. As PCSK9 inhibitors have been demonstrated to significantly and safely reduce LDL-C, discussions have begun to explore its optimal timing in coronary artery disease, especially in subjects with acute coronary syndrome (ACS). Also, their additional benefits, such as anti-inflammatory effects, plaque regression effects, and cardiovascular event prevention, have become the focus of recent research. Several studies, including EPIC-STEMI, suggest the lipid-lowering effects of early PCSK9 inhibitors in ACS patients, while some studies such as PACMAN-AMI suggest that early PCSK9 inhibitors can decelerate plaque progression and reduce short-term risks of cardiovascular events. Thus, PCSK9 inhibitors are entering the era of early implementation. In this review, we are committed to summarizing the multidimensional benefits of early implementation of PCSK9 inhibitors in ACS.

## Introduction

With the progress of medical technology, the prognosis of patients with coronary heart disease, the leading cause of death in humans today, has been improved, while there is scope to optimize strategies for preventing and treating coronary heart disease (1, 2). Hyperlipidemia, especially lower-density lipoprotein cholesterol (LDL-C), is one of the major risk factors for atherosclerotic cardiovascular disease (ASCVD) (3). Total LDL-C exposure is positively correlated with the incidence of clinical events, and the risk of cardiovascular events is reduced by approximately 22% for every 1 mmol/l decrease in LDL-C (4, 5).

Proprotein convertase subtilisin/kexin type 9 (PCSK9) inhibitors are now important lipid-lowering drugs in addition to the classic lipid-lowering drugs statins and ezetimibe since they can reduce the degradation of LDL receptors and increase the clearance of LDL-C (6–8). Currently, PCSK9 monoclonal antibodies (PCSK9-mAbs), including evolocumab (Amgen) and alirocumab (Sanofi), are the most commonly used PCSK9 inhibitors in clinical practice.

In addition to lowing LDL-C, PCSK9 inhibitors also decrease other lipid levels such as apolipoprotein (apo) B, lipoprotein(a) [Lp(a)], and non-high-density lipoprotein cholesterol (non-HDL-C), stabilize the plaque, inhibit inflammation and reduce the risk of cardiovascular events (9–11). Based on the above multi-dimensional therapeutic effect, several clinical studies have shown that the implementation of PCSK9 inhibitors greatly reduces lipid levels and the occurrence of cardiovascular adverse events in patients with coronary heart disease (10, 12). Besides, recent studies suggest that early implementation of PCSK9 inhibitors also have significant benefits of lipid lowing and plaque regression in ACS patients (13–15). Therefore, the timing of PCSK9 inhibitors for ACS advancing from the failure to achieve LDL-C goal after 4–6 weeks recommended by recent guidelines to “pre or post PCI implementation” (16). PCSK9 inhibitors are now entering the era of early implementation. Herein, we reviewed the role of early application of PCSK9 inhibitors in patients with coronary heart disease, especially those with ACS, by summarizing clinical trials and meta-analyses in recent years. These sufficient evidences provide theoretical basis for clinical practice of early implementation of PCSK9 inhibitors in ACS.

## Methods

The keywords “PCSK9 inhibitors”, “PCSK9 mAbs”, “evolocumab” and “alirocumab” were used to search for literature on databases including PubMed, Web of Science, Google Scholar and ClinicalTrial.gov. The trials of early initiation of PCSK9 inhibitors in ACS were included in this study. In addition, some of the recent studies have been accessed through academic conferences.

## Lipid-lowering effect of PCSK9 inhibitors and early implementation

**Figure 1:**
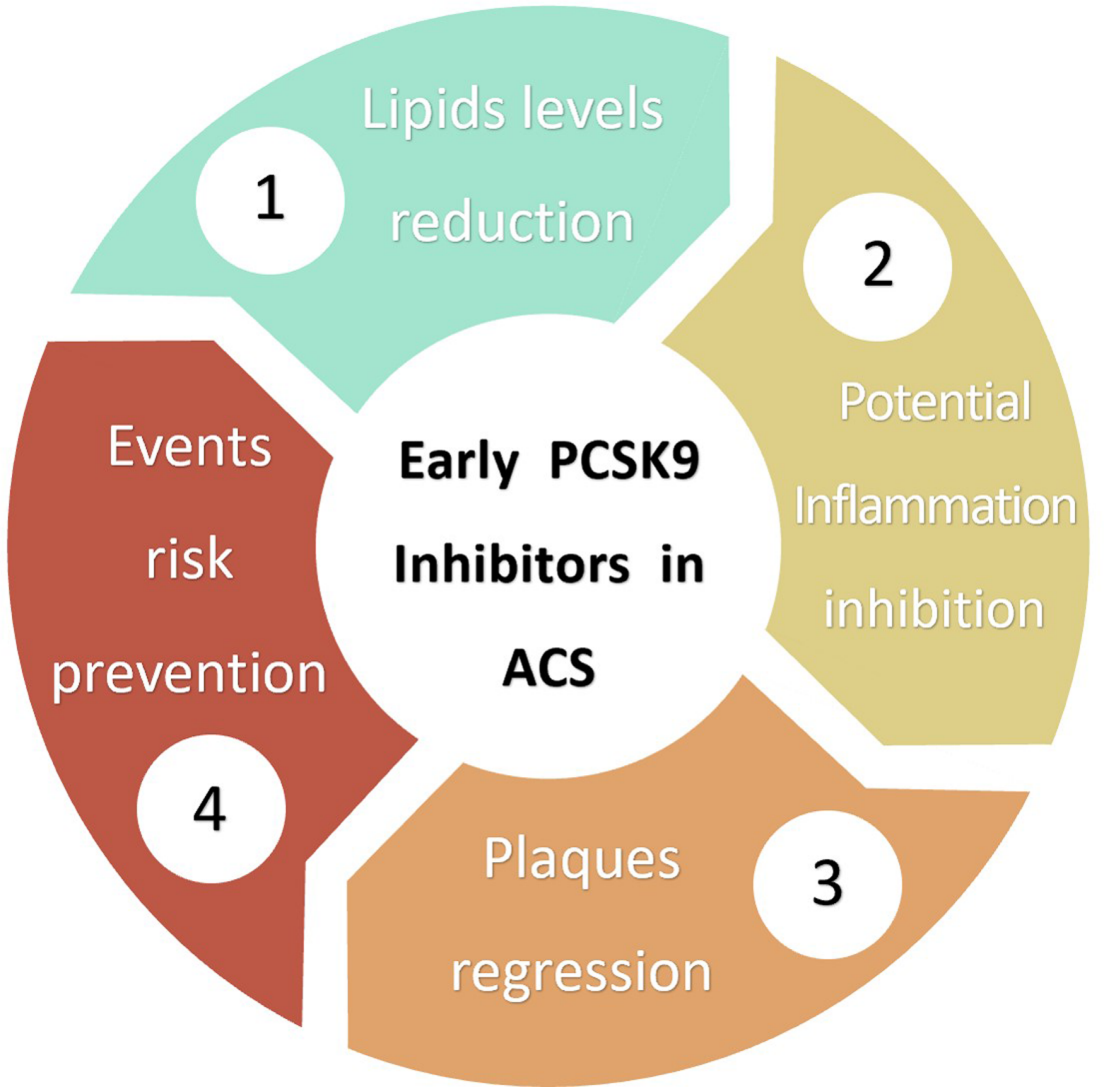
Benefits and potential benefits of early PCSK9 inhibitors in ACS.

LDL-C reduction is the most significant benefits of PCSK9 inhibitors (Figure 1). Numerous studies, including the Odyssey Outcomes study (*n* = 18,924) and the Fourier study (*n* = 27,564), show that in patients receiving statins therapy, PCSK9 inhibitors resulted in an approximately 50%–63% reduction in LDL-C (17–19). In addition, a meta-analysis by Yi-Ting Huang et al. (*n* = 42,786) revealed that PCSK9 inhibitors reduced LDL-C by 68.05%, apo B by 54.95%, and Lp(a) by 34.25% (20). Zhang Yue et al*.* conducted a meta-analysis included 14 studies (*n* = 52,586) to show that PCSK9 inhibitors significantly reduced LDL-C, total cholesterol, triglyceride, Lp(a), non-HDL-C and apo B and increased HDL-C and apoA1 levels (21). Another meta-analysis by Farmakis et al*.* (*n* = 64,107) showed that PCSK9 inhibitors reduced Lp(a) levels by 26.7% and reduced LDL-C by 54% (22). Beyond its powerful lipid-lowering effects, PCSK9 inhibitors has a rapid onset of action to reduce circulating PCSK9 levels by approximately 97% within 24 h of implementation. Meanwhile, most patients achieved low LDL-C levels within the first or second month of dosing, which provides a theoretical basis for the early implementation of PCSK9 inhibitors (23–25).

EVOPACS was a randomized, double-blind, placebo-controlled multicenter clinical study that involved 308 patients [evolocumab (*n* = 155) or placebo (*n* = 153)] with new-onset ACS who did not meet or were not expected to achieve lipid goals and was first to demonstrate the lipid-lowering effectiveness of early PCSK9 inhibitors in patients with ACS (14). In addition to statin treatment, the trial group was given evolocumab (420 mg monthly) as early as possible (within ≤24 h) following randomization, while the matching placebo was given to the control group (14). The mean LDL-C level reduced from 3.61 mmol/l to 0.79 mmol/l in the trial group and from 3.42 mmol/l to 2.06 mmol/l in the placebo group after eight weeks. Furthermore, the total cholesterol level decreased by 26.5%, apolipoprotein B by 34.2%, and non-HDL-C by 34.6% after eight weeks in the trial group compared to the placebo group (14).

EPIC-STEMI (*n* = 68) was a randomized, double-blind, placebo-controlled clinical study of ST-segment elevation myocardial infarction (STEMI) patients without baseline LDL or statin use routinely assessed and undergoing immediate PCI (26). The PCSK9 inhibitors significantly reduced lipid levels in the trial group (alirocumab 150 mg bi-weekly) compared to the control group, indicating that early PCSK9 inhibitors effectively reduce LDL-C levels regardless of baseline LDL-C levels and basic statin therapy (26).

The EVACS series of studies involved 100 subjects with ACS (27, 28). The EVACS I study included 57 NSTEMI patients receiving high-intensity statin therapy who were treated with PCSK9 inhibitors within 24 h (the test group).The participants in the test group had significantly decreased LDL-C levels on the first day (from 91.5 ± 35 mg/dl to 70.4 ± 27 mg/dl), further reducing by 28.6 mg/dl after 30 days compared to the control group. Furthermore, another observational study involved patients from EVACS I and EVACS II found that the Lp(a) levels in 74 of these acute myocardial infarction (AMI) patients were elevated in the placebo group compared to baseline, suggesting that PCSK9 inhibitors may inhibit the production of Lp(a) during AMI onset (27, 28).

VCU-AlirocRT involved 20 patients with new-onset non-STEMI (NSTEMI) who were receiving high-intensity statin therapy randomized into two groups (*n* = 20) within 24 h of presentation, with one injection of alirocumab 150 mg in the test group and the matching placebo in the control group (29). Alirocumab treatment significantly reduced LDL-C from baseline to 14 days by 64 mg/dl (−96, −47) compared to placebo [+1 mg/dl, (−25, + 16)] (29).

Taken together, these results suggest that early PCSK9 inhibitors can rapidly reduce lipid levels, implying that patients with ACS can benefit from and achieve low lipid levels with early PCSK9 inhibitor treatment (Figure 1, Table 1).

**Table 1:** Studies that have evaluated the effects of PCSK9-mAbs.

Study	Study Population (*n*)	Treatment	Primary Outcome
EVOPACS KOSKINAS KC et al. (2019) (14)	308	Evolocumab 420 mg monthly vs. placebo	Significant reduction in several lipids levels; No effects in inflammatory indicators
EPIC-STEMI MEHTA SR et al. (2022) (26)	68	Alirocumab 150 mg bi-weekly vs. placebo	Alirocumab reduced LDL-C by 22% compared with sham control before primary PCI in STEMI patients
EVACS I LEUCKER TM et al. (2020) (27)	57	Evolocumab 420 mg monthly vs. placebo	LDL-C in the evolocumab participants was on average 28.6 mg/dl lower than in the placebo participants at 30 days
VAVURANAKIS MA et al. (2020) (28)	74	Evolocumab 420 mg monthly vs. placebo	Evolocumab within 24 h of hospital admission prevents Lp(a) elevation during the peri-infarct and early post-infarction period in AMI patients
VCU-AlirocRT TRANKLE CR et al. (2019) (29)	20	Alirocumab 150 mg once vs. placebo	Lipids levels reduced at 72 h and 2 weeks
Ye-Xuan Cao et al. (2018) (30)	4,198	PCSK9-mAbs	No changes in hs-CRP
Amirhossein Sahebkar et al. (2022) (31)	87,669	PCSK9-mAbs	Significant decrease in LDL-C level; No changes in hs-CRP; No significant differences in the risks of cardiovascular death, myocardial infarction, and stroke compared with the statins group and ezetimibe group
Wenjia Yang et al. (2016) (32)	2,546	PCSK9-mAbs	No changes in hs-CRP
GLAGOV NICHOLLS SJ et al. (2016) (33)	968	Evolocumab 420 mg monthly vs. placebo	Evolocumab resulted in a greater decrease in percent atheroma volume and total atheroma volume
PACMAN-AMI RABER L et al. (2022) (15)	300	Alirocumab 150 mg bi-weekly vs. placebo	Alirocumab resulted in greater coronary plaque regression at 52 weeks among AMI patients
HUYGENS NICHOLLS SJ et al. (2022) (34)	150	Evolocumab 420 mg monthly vs. placebo	Evolocumab demonstrated a greater increase in minimum fibrous cap thickness and decrease in maximum lipid arc and macrophage index
Wang Zhe et al. (2022) (35)	65	Evolocumab 140 mg once vs. placebo	Evolocumab increased TMPG indexes and decreased CTFC indexes after PCI and 30 days after PCI among STEMI patients
FOURIER-OLE O'DONOGHUE M L et al. (2022) (36)	6,635	Evolocumab 140 mg bi-weekly or 420 mg monthly vs. placebo	Timely application of evolocumab was associated with lower rates of adverse events compared with delayed treatment initiation
Yan Hao et al. (2022) (37)	136	Evolocumab 140 mg bi-weekly vs. placebo	Evolocumab reduced lipids levels and the incidence of MACEs in patients with extremely high-risk ACS

## Early PCSK9 inhibitors and inflammatory indicators

Inflammation plays a key role in the development and progression of ACS, while anti-inflammatory therapy remains controversial (38). PCSK9, as a trigger for the expression of pro-inflammatory cytokines, is significantly elevated during ACS (23, 39). Several studies found that it was positively associated with the risk of cardiovascular events, which may be related to multiple mechanisms, such as inflammatory cascade activation and additional platelet release and aggregation (40–42). Early PCSK9 inhibitor treatment can potentially inhibit PCSK9-related inflammatory effects in patients with ACS. Accordingly, some studies have analyzed data on inflammatory indicators of PCSK9 inhibitors for ACS to explore their anti-inflammatory benefits (Table 1).

EVOPACS analyzed inflammatory indicators such as high sensitivity C- reactive protein (hs-CRP), Interleukin (IL-) 1β, and IL-6 showing no difference at week 4 between groups, suggesting that early PCSK9 inhibitor treatment may not significantly reduce inflammatory indicators (14). Furthermore, meta-analyses by Ye-Xuan Cao et al. (*n* = 4,198), Amirhossein Sahebkar et al. (*n* = 87,669) and Wenjia Yang et al. (*n* = 2,546) suggested that PCSK9 inhibitors did not significantly reduce hs-CRP levels in the short term, regardless of the type of PCSK9 inhibitor or the frequency of dosing (30–32).

Although previous animal models have confirmed that PCSK9 might exert inflammatory effects, PCSK9 inhibitors have failed to significantly reduce circulating systemic markers such as hs-CRP, interleukin and TNF-α in ACS patients based on this evidence (43–47). Considering that part of the beneficial influence of PCSK9 inhibitors is related to their ability to attenuate low-grade systemic inflammation, implementation of PCSK9 inhibitors in ACS patients may fail to inhibit high-grade inflammatory cascade responses, which may account for this phenomenon (48, 49). Further exploration of the anti-inflammatory mechanisms of PCSK9 inhibitors is required.

## Early PCSK9 inhibitors and coronary imaging changes

The effects of PCSK9 inhibitors on plaques have been the focus in the context of the widespread use of lipid-lowering therapy in coronary artery disease. The GLAGOV study (*n* = 968) suggested that PCSK9 inhibitors could promote regression in plaque composition (33, 50). Studies are underway to determine the effects of the early implementation of PCSK9 inhibitors on plaque, blood flow, vascular inflammation, and other relevant imaging features in patients with ACS (13, 15, 34, 35, 51).

PACMAN-AMI was a multicenter, double-blind, placebo-controlled, randomized trial involving 300 AMI patients with the basic treatment of high intensity statins who initiated PCSK9 inhibitors less than 24 h after an urgent PCI of the culprit lesion (15, 51). The plaque condition was assessed by imaging techniques such as IVUS, OCT, and near-infrared spectroscopy showing that at 52 weeks, the mean change in the percentage of atherosclerotic volume was −2.13% in the alirocumab group and −0.92% in the placebo group [difference −1.21% (95% CI, −1.78% to −0.65%)], and the maximum lipid core burden index within 4 mm decreased by 79.42 in the alirocumab group and by 37.60 in the placebo group [difference 41.24 (95% CI, −70.71 to −11.77)], while the mean change in the minimum fibrous cap thickness was 62.67 μm in the alirocumab group and 33.19 μm in the placebo group [difference 29.65 μm (95% CI, 11.75–47.55)] (15, 51). These results suggest that early PCSK9 inhibitors in AMI patients stabilizes coronary plaques and reduces the intraplaque lipid load.

HUYGENS was a phase 3, multicenter, double-blind, randomized controlled study that enrolled 150 patients with non-ST-segment elevation ACS who were randomized 1:1 to the trial group (evolocumab 420 mg administered by subcutaneous injection monthly for 48 weeks) and the control group (matching placebo) after PCI (34). The primary study endpoint was the minimum fibrous cap thickness measured by OCT at 50 weeks, with a greater increase in the evolocumab group (+42.7 vs.+ 21.5 μm). Furthermore, there was a decrease in the maximum lipid arc (−57.5° vs. −31.4°) and macrophage index (−3.17 vs. −1.45 mm) in the trial group, indicating that early PCSK9 inhibitor treatment in ACS patients is beneficial for plaque stabilization and regression (34).

Another study by Wang Zhe et al. involved 65 STEMI patients, and divided them into the test group (loading dose of statins combined with PCSK9 inhibitors, 35 patients) and the control group (loading dose of statins only, 30 patients) (35). The corrected TIMI frame count (CTFC) index was significantly lower in the test group immediately after PCI and 30 days after PCI, while the TIMI myocardial perfusion grading (TMPG) index was higher than in the control group (35). However, the treatment did not decrease the incidence of cardiovascular death, non-fatal myocardial infarction, or target vessel revascularization (35).

The available findings suggest that PCSK9 inhibitors have beneficial effects on improving plaque volume and stability, as well as reducing lipid load, providing new evidence for implementing PCSK9 inhibitors during ACS (Figure 1). It is anticipated that ongoing studies such as INTENSITY-HIGH (*n* = 60), ANTARES (*n* = 30), ASAP-SVG (NCT03542110), MICROPROTECT (NCT04338165), YELLOW III (NCT04710368), etc., will provide further evidence (52–54).

## Research advances in clinical events of PCSK9 inhibitors

The main findings of ODYSSEY OUTCOMES and FOURIER were that PCSK9 inhibitors could reduce the risk of long-term cardiovascular events (ODYSSEY OUTCOMES ([9.5%] vs. [11.1%]; HR 0.85; 95% CI, 0.78 to 0.93) and FOURIER ([9.8%] vs. [11.3%]; HR 0.85; 95% CI, 0.79 to 0.92) (17, 18). Considering that PCSK9 levels increase significantly in the early stages after ACS, early PCSK9 inhibitor treatment is expected to reduce the short-term risk of cardiovascular events in ACS patients (Figure 1).

FOURIER-OLE involved 6,635 patients derived from the parent study FOURIER randomized 1:1 to the test group (subcutaneous injection of evolocumab 140 mg every two weeks or 420 mg every month) and the control group (matching placebo) (36). After a median follow-up of 5 years and a maximum follow-up of 8.4 years, the risk of cardiovascular death, myocardial infarction, stroke, and hospitalization for unstable angina or coronary revascularization was reduced by 15% (HR 0.85 [95% CI 0.75–0.96]) in the test group, with a 20% reduction in the risk of cardiovascular death, myocardial infarction, or stroke [HR 0.80 (0.68–0.93)] and 23% reduction in the independent risk of death [HR 0.77 (0.60–0.99)], and the benefit was greater in the first three years. FOURIER-OLE further demonstrates the beneficial effects and favorable safety profile of PCSK9 inhibitors in preventing the risk of cardiovascular events and recommends early implementation of PCSK9 inhibitors based on the benefit curve.

A randomized controlled study by Yan Hao et al. involved 136 ACS patients who were stratified as very high-risk for lipid management (37). The primary endpoint was major adverse cardiovascular events (MACE) at three months, and early PCSK9 inhibitor treatment after PCI in ACS patients resulted in a significant decrease in MACE at three months (8.82% vs. 24.59%).

PCSK9 plays an important physiological role in metabolism, and low lipid levels are considered a risk factor for hemorrhagic stroke, therefore, the safety of PCSK9 inhibitors was previously controversial (55, 56). Classical clinical trials, including the Odyssey outcomes, Fourier, Ebbinghaus and Descartes results, suggested that PCSK9 inhibitors may only lead to an increased risk of injection-site reactions, while not suggesting that PCSK9 inhibitors may cause other adverse events such as diabetes and neurocognitive dysfunction (17, 18, 57, 58). Recent studies including EVOPACS, EPIC-STEMI and PACMAN-AMI, suggest that PCSK9 inhibitors does not significantly increase the risk of multiple adverse events in ACS patients (14, 15, 26, 34). Furthermore, several meta-analyses conducted by Qiwen Chen et al*.* (*n* = 65,957), Hangying Ying et al. (*n* = 128,691) and Hirsh R B et al. (*n* = 59,733) suggest that PCSK9 inhibitors and low LDL-C levels do not increase the additional risk of neurocognitive adverse events and new onset diabetes (59–61).

The available evidence suggests that early PCSK9 inhibitor treatment may have potential benefits in short-term events prevention, while the risks of adverse events are in line with expectations. More evidence is urgently needed, and more attention should be paid to the sample size and baseline control of relevant studies, as many factors may contribute to adverse events in the acute phase of ACS.

## Discussion

**Figure 2:**
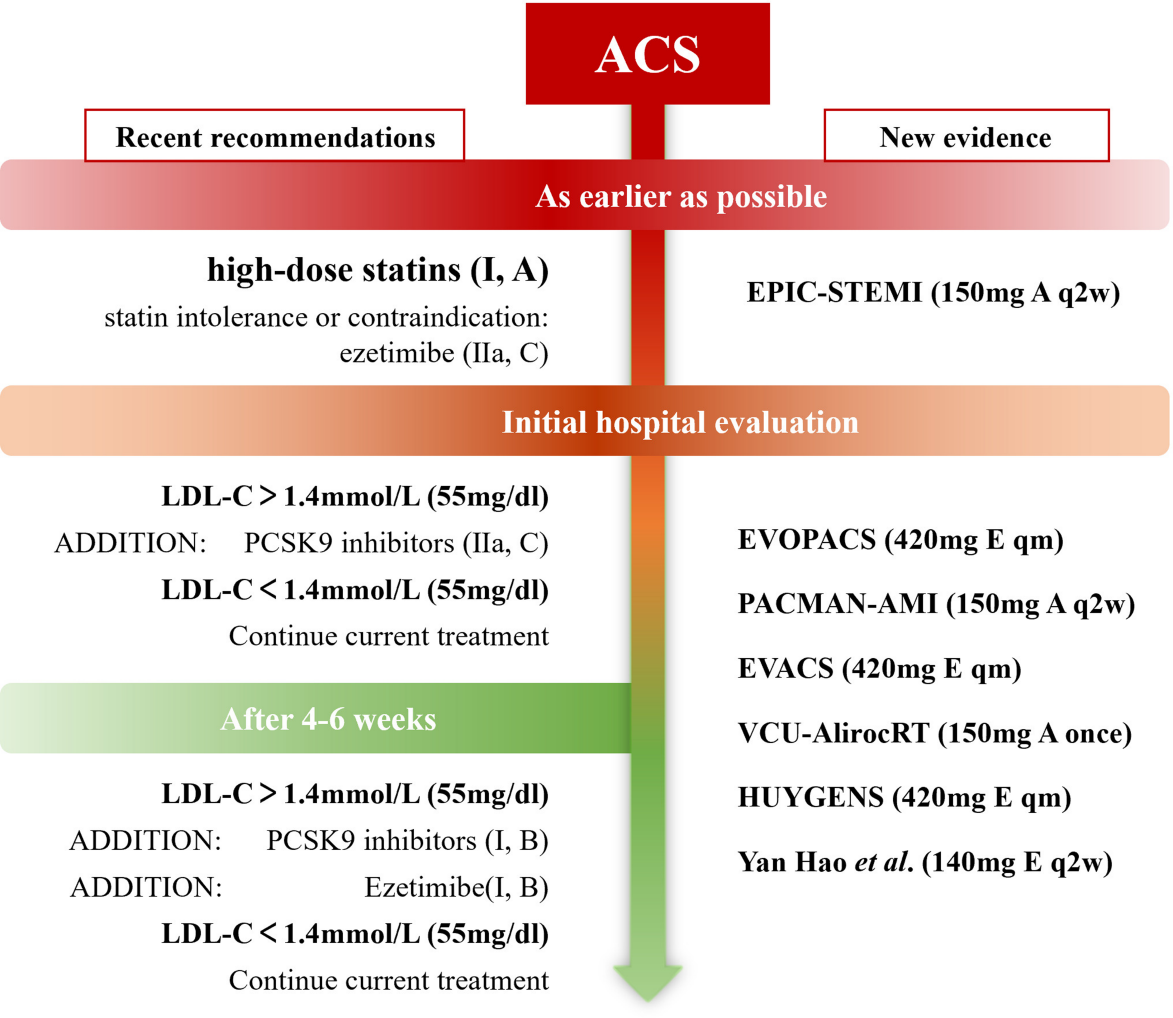
Recent recommendations and new evidence on the timing of PCSK9 inhibitor initiation (New evidence: A: alirocumab; E: evolocumab).

PCSK9 inhibitors are the most potent lipid-lowering therapies available, and they have been used for several years as an adjunct to lipid management in patients with coronary artery disease. New evidence on the multidimensional benefits, timing of use, and appropriate population of PCSK9 inhibitors has been added from recent studies. Current guidelines recommend PCSK9 inhibitors in patients whose LDL-C goals are not achieved after 4–6 weeks (Figure 2) (16, 62). New evidence of on the multidimensional benefits, timing of use, and appropriate population of PCSK9 inhibitors has been added from recent clinical studies.

Application of PCSK9 inhibitors within 4 weeks of the presentation of typical ACS symptoms is considered early implementation. In the phase of ACS, elevated PCSK9 may lead to a diminished effect of LDL-R and increased LDL-C levels while promoting inflammatory responses and plaque progression. Based on the above mechanism, early PCSK9 inhibitors treatment has been implemented as an aggressive therapy in ACS patients, and could significantly lower lipid in the treatment group regardless of LDL-C levels (26).

A number of clinical trials, including EVOPACS, have demonstrated the efficacy of early PCSK9 inhibitors. The EPIC-STEMI trial implemented a protocol for preoperative application of PCSK9 inhibitors that not only provided evidence for earlier initiation of PCSK9 inhibitors, but also explored the feasibility of a study design using non-fasting lipids as baseline lipids. In contrast to the current research on the inflammatory mechanisms of PCSK9, although plasma PCSK9 levels are significantly elevated during ACS, studies covered in this reviews suggest that early implementation of PCSK9 inhibitors does not reduce circulating markers of inflammation. However, there is evidence, e.g., from ALTAIR (*n* = 24), that long-term application of PCSK9 inhibitors may lead to a reduction in macrophage grade (63). Further research advances on the inflammatory benefits of PCSK9, such as new inflammatory markers associated with PCSK9, are expected to guide subsequent clinical trials and clarify the potential anti-inflammatory benefits of early PCSK9 inhibitors. Studies also suggest that early implementation of PCSK9 inhibitors may be associated with intraplaque inflammation, coronary plaque regression and increases in TMPG levels. These studies illustrate the benefits of early PCSK9 inhibition in terms of plaque regression and hemodynamic stabilization. Yan Hao et al. showed a significant reduction in MACE events in patients treated with early PCSK9 inhibitors, which has not been shown in other studies (37). This indicates that early PCSK9 inhibitors may provide a clear but modest theoretical benefit in reducing short-term cardiovascular risk. In addition, some studies have also suggested that early PCSK9 inhibitors may lead to several mild adverse effects such as injection-site reactions (17, 18, 64).

There are two dimensions taken by most recent studies. The first dimension is about the beneficial effects of early PCSK9 inhibitors independent of the lipid-lowering effects, such as coronary plaque and blood flow improvement presented on general coronary angiography and optical coherence tomography (OCT). Such studies provide evidence of clinical benefits on multiple dimensions of PCSK9 inhibitors, but the small sample sizes are a major limitation due to the higher demand for imaging data and the difficult acceptance of frequent coronary angiograms and other imaging examinations. The second dimension relates to the optimal timing of PCSK9 inhibitors. Even though there is emerging evidence to support the early implementation of PCSK9 inhibitors, the optimal timing of their initiation in patients with different clinical characteristics remains unclear. Considering the urgent treatment process, conducting clinical trials on the use of PCSK9 inhibitors before PCI in ACS remains challenging.

Overall, the efficacy and safety of early PCSK9 inhibitors have been supported by increasing evidence and their limited drawbacks should not be overly considered, and we therefore believe that the establishment of better lipid management strategies for patients with ACS is essential. It is foreseeable that as time advances and guidelines are updated, early PCSK9 inhibitor treatment is likely to be widely adopted in the future as a basic therapy for lipid management in patients with ACS.

## Conclusion

The current evidence has demonstrated the benefits of early PCSK9 inhibitors in lipid reduction, plaque stabilization, and short-term or long-term cardiovascular event prevention. Furthermore, most adverse effects resulting from early PCSK9 inhibitors are mild and manageable, consistent with long-term follow-up results. However, further evidence is needed to support the anti-inflammatory effects and cardiovascular events prevention benefits of early PCSK9 inhibitors.
